# Respiratory support in COPD patients after acute exacerbation with monitoring the quality of support (Rescue2-monitor): an open-label, prospective randomized, controlled, superiority clinical trial comparing hospital- versus home-based acute non-invasive ventilation for patients with hypercapnic chronic obstructive pulmonary disease

**DOI:** 10.1186/s13063-020-04672-w

**Published:** 2020-10-22

**Authors:** On behalf of the Rescue2-monitor group, J. Gonzalez-Bermejo, D. Hajage, I. Durand-Zaleski, J. M. Arnal, A. Cuvelier, L. Grassion, S. Jaffre, B. Lamia, S. Pontier, A. Prigent, C. Rabec, C. Raherison-Semjen, C. Saint Raymond, J. Soler, W. Trzepizur, J. C. Winck, M. Aguiar, H. Chaves, B. Conde, M. J. Guimarães, P. Lopes, A. Mineiro, S. Moreira, P. Pamplona, C. M. Rodrigues, S. Sousa, A. Antón, A. Córdoba-Izquierdo, C. Embid, C. Esteban González, F. Ezzine, P. Garcia, M. González, I. Guerassimova, D. López, M. Lujan, S. Martí Beltran, J. M. Martinez, F. Masa, N. Pascual, N. Peñacoba, P. Resano, L. Rey, F. Rodríguez Jerez, A. Roncero, Je. Sancho Chinesta, Ja Sayas Catalán

**Affiliations:** 1 Gonzalez-Bermejo, J. Sorbonne Université, INSERM, UMRS1158 Neurophysiologie Respiratoire Expérimentale et Clinique, 75005 Paris, France; 2 AP-HP, Groupe Hospitalier Universitaire APHP-Sorbonne Université, site Pitié-Salpêtrière, Service de Pneumologie, Médecine Intensive et Réanimation (Département R3S), 75013 Paris, France

**Keywords:** Chronic obstructive pulmonary disease (COPD), Non-invasive ventilation (NIV), Hypoventilation, Targeted treatment, Hypercapnic respiratory failure

## Abstract

Chronic obstructive pulmonary disease (COPD) is expected to be the 3^rd^ leading cause of death worldwide by 2020. Despite improvements in survival by using acute non-invasive ventilation (NIV) to treat patients with exacerbations of COPD complicated by acute hypercapnic respiratory failure (AHRF), these patients are at high risk of readmission and further life-threatening events, including death. Recent studies suggested that NIV at home can reduce readmissions, but in a small proportion of patients, and with a high level of expertise. Other studies, however, do not show any benefit of home NIV. This could be related to the fact that respiratory failure in patients with stable COPD and their response to mechanical ventilation are influenced by several pathophysiological factors which frequently coexist in the same patient to varying degrees. These pathophysiological factors might influence the success of home NIV in stable COPD, thus long-term NIV specifically adapted to a patient’s “phenotype” is likely to improve prognosis, reduce readmission to hospital, and prevent death. In view of this conundrum, Rescue2-monitor (R2M), an open-label, prospective randomized, controlled study performed in patients with hypercapnic COPD post-AHRF, will investigate the impact of the quality of nocturnal NIV on the readmission-free survival. The primary objective is to show that any of 3 home NIV strategies (“rescue,” “non-targeted,” and “targeted”) will improve readmission-free survival in comparison to no-home NIV. The “targeted” group of patients will receive a treatment with personalized (targeted) ventilation settings and extensive monitoring. Furthermore, the influence of comorbidities typical for COPD patients, such as cardiac insufficiency, OSA, or associated asthma, on ventilation outcomes will be taken into consideration and reasons for non-inclusion of patients will be recorded in order to evaluate the percentage of ventilated COPD patients that are screening failures. ClinicalTrials.gov NCT03890224. Registered on March 26, 2019.

## Background

According to the Global Burden of Disease (GBD), chronic obstructive pulmonary disease (COPD) is the third leading cause of death worldwide, something that WHO had not predicted to occur until 2030 [1] and is expected to become the leading cause of death in the next 15 years [2]. Respiratory failure due to severe acute exacerbations is now recognized as an independent negative prognostic factor with mortality increasing with the frequency of severe exacerbations.

Respiratory failure in patients with stable COPD and their response to mechanical ventilation are influenced by several pathophysiological factors, such as ventilation/perfusion ratio, obesity, and its relation to hypoventilation, muscle function/myopathy, and comorbidities, such as obstructive sleep apnea hypopnea syndrome (OSAHS) and/or chronic heart failure [3]. Thus, every patient is different, because many of these factors coexist in the same patient to varying degrees.

Although there is no evidence that “targeted” treatments are more effective, it follows that the success of home non-invasive ventilation (NIV) in stable COPD may be largely influenced by the main underlying pathophysiological process. Therefore, long-term home NIV, specifically adapted to the patients “phenotype”, is likely to improve prognosis, reduce readmission to hospital or death in COPD patients who remain persistently hypercapnic, and thus result in a more equilibrated clinical situation for the patients.

Already in the 1960s, clinicians speculated that home intermittent respiratory muscle rest afforded by negative-pressure ventilators might also benefit daytime respiratory muscle performance of patients with severe COPD [4]. This approach was, however, generally abandoned in the early 1990s after a large randomized clinical trial nocturnally using a “jacket” negative-pressure ventilator showed no improvement in exercise endurance or respiratory muscle strength in patients with severe COPD, and acceptance of the device by patients was poor [5]. Subsequent studies using home NIV have reported conflicting results with negative findings [6, 7], a slight improvement in quality of life [8], improvements in dyspnea and sleep [9], or reduced mortality, but in association with worse quality of life [10]. Thus, the 2013 Cochrane review of long-term NIV for COPD concluded that there was no evidence of significant benefit in any of the measured indices [11].

Despite the improvements in survival by using acute NIV to treat patients with exacerbations of COPD complicated by acute hypercapnic respiratory failure (AHRF), these patients are at high risk of readmission and further life-threatening events [12, 13]. In a recent study of 110 patients with AHRF (RESCUE study), 65% had another life-threatening event and 49% had died within 1 year after discharge [14].

More recently, however, Köhnlein et al. [15] found a significant reduction of mortality in the treatment arm, and Murphy et al. [16] reported a significant improvement in time to readmission or death (from 1.4 to 4.3 months) and concomitant reduction of 1-year risk of readmission or death by 17% (from 80.4 to 63.4%) for the group receiving home NIV. Additionally, the home NIV group reported a higher quality of life at 3 months, leading the authors to recommend the consideration of home NIV for patients with severe COPD and persistent hypercapnia after a life-threatening exacerbation, but in a very few proportion of patients (6% of the screened patients). Furthermore, the Global initiative for Obstructive Lung Disease (GOLD) 2017 document [17] indicated that home NIV may be considered of some use only in a selected group of patients, particularly in those with pronounced daytime hypercapnia and recent hospitalization.

In summary, home NIV is a complex therapy that has been shown to be of benefit for a number of chronic conditions. In stable COPD, however, the evidence is contradictory and home NIV is currently only suggested for very severe and rare and selected patients [15, 16]. Thus, home NIV in patients with stable COPD continues to be a source of controversy with the concomitant urgent need to develop strategies to reduce the number and severity of exacerbations of COPD.

Currently, two main restraints hamper the development of such strategies, (i) the lack of systematic diagnostic approaches in order to define the underlying pathophysiological phenotype and (ii) the lack of clinical trials on the impact of home NIV in conjunction with pathophysiological phenotypes and their influence on the efficacy/safety ratio. Furthermore, periodical evaluation of compliance, tolerance, and efficacy should be consensual to ensure an adequate delivery of home NIV.

Thus, in order to optimize long-term home NIV, further research is required to identify responders, the relevance of hypercapnic status change in clinical outcomes, the optimum time points for starting home NIV, and equipment settings or hours of ventilation to be efficient. With healthcare objectives and budget constraints, telemonitoring of COPD patients is an important challenge in most European countries.

Therefore, the primary objective of Rescue2-monitor is to test the superiority of any of 3 modalities of home NIV compared to a standard treatment without home NIV (control group) in terms of improving admission-free survival of persistently hypercapnic COPD patients. Furthermore, home NIV with a highly targeted ventilator strategy (“TARGETED HOME VENTILATION” with high level of phenotyping of the patient and high level of monitoring of the quality of the home NIV) will be compared to the other three groups. For the adaptation of this targeted home ventilatory strategy, three main phenotypes are considered as determining factors, (i) OSAHS, (ii) heart failure, and (iii) diaphragmatic dysfunction. For patients without any of these phenotypes, the targeted home ventilatory strategy will be adapted with a high level of monitoring of the quality and purpose of the different settings of the ventilator.

## Methods/design

### Aim and objectives

#### Primary objective

**Fig. 1:**
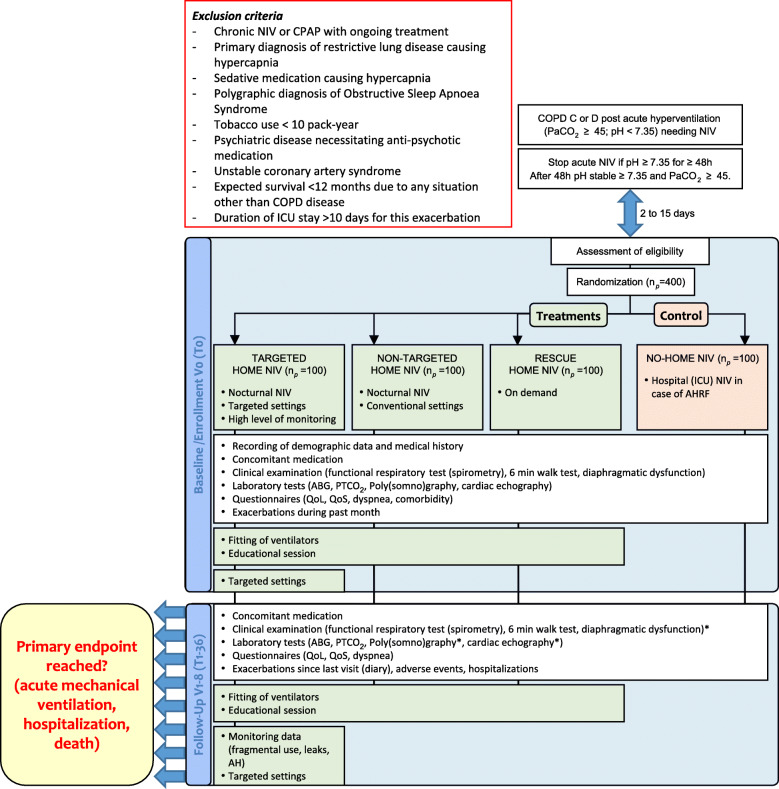
Flow diagram of study procedure: *n*_p_ = planned number of patients, V1 to 8 = visit number, T0 to 36 = time in months. For the follow-up visit, the (*) indicates evaluations that are performed if not done previously (at baseline) or if a follow-up is necessary due to abnormal results. Independently of abnormal results, spirometry will be repeated at V2 (T3) or V3 (T6)

The primary objective of this study is to demonstrate the superiority of any of the three home NIV strategies over standard no-home NIV (only hospital NIV in case of acute respiratory failure) in terms of admission-free survival of COPD patients after an episode of acute respiratory insufficiency with hypoventilation (AHRF) (Fig. 1).

#### Secondary objectives

The secondary objectives of the study are to demonstrate for severe COPD patients after an episode of acute respiratory insufficiency with hypoventilation, that any of the three home NIV strategies are superior to standard no-home NIV in terms of:

- Global survival
- Exacerbations frequency
- Comorbidity
- Questionnaires (quality of life, sleep, and dyspnea)
- (Serious) adverse event (AEs and SAEs) occurrence rate
- Cost-effectiveness

### Study design

Rescue2-monitor is an open-label, prospective randomized, controlled, superiority clinical trial, performed in patients with hypercapnic chronic obstructive pulmonary disease post-acute hypercapnic exacerbation (ClinicalTrials.gov Identifier: NCT03890224).

**Fig. 2:**
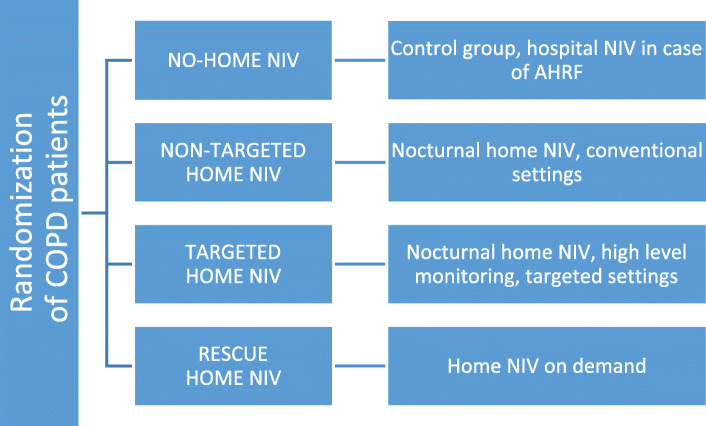
Study design. Treatment arms are randomized in a 1:1:1:1 ratio

Four arms (randomization to groups in a ratio of 1:1:1:1) will be compared, i.e., no-home NIV (hospital NIV/control group) versus any of 3 modalities of nocturnal home NIV (test groups). The 3 test treatments are non-targeted home NIV, targeted home NIV, and rescue home NIV (Fig. 2).

### Study setting

The study is an international multicenter study involving 13 hospitals in France, 20 hospitals in Spain, and 8 hospitals in Portugal. Further, centers are envisaged in Italy. Within a recruitment period of 2 years, a total of 400 patients (100 patients per treatment arm) will be recruited during standard hospitalization after an acute exacerbation (between 2 and 15 days after the acute exacerbation).

### Study population

COPD patients will be recruited during standard hospitalization after an acute exacerbation, based on the following criteria:

#### Inclusion criteria

- Patients with COPD, GOLD C or D, and forced expiratory volume in 1 s (FEV1) < 65%
- ARHF (pH < 7.35 and PaCO_2_ ≥ 45 mmHg (≥ 6 kPa) treated more than 24 h with ventilation (non-invasive or invasive)
- 48 h to 2 weeks with pH > 7.35, and PaCO_2_ ≥ 45 (≥ 6 kPa) after NIV withdrawal, during daytime at rest without oxygen or ventilatory support (or with O_2_ if patients are not able to avoid O_2_ with immediate desaturation below 80%)

#### Exclusion criteria

- Patient treated with chronic NIV or CPAP, with ongoing treatment
- Primary diagnosis of restrictive lung disease causing hypercapnia, i.e., obesity hypoventilation and chest wall disease; however, these patients will be included if the FEV1/FVC ratio is < 60% and the FEV1 < 50% if the predominant defect is considered to be obstructive by the center clinician
- BMI > 35 kg/m^2^
- Sedative medication causing hypercapnia (> 3 drugs or more than 20 mg of morphine/day)
- Polygraphic diagnosis of obstructive sleep apnea syndrome (AHI > 30/h)
- Cognitive impairment that would prevent informed consent
- Pregnancy
- Tobacco use < 10 pack-year
- Psychiatric disease necessitating anti-psychotic medication, ongoing treatment for drug or alcohol addiction, persons of no fixed abode post-discharge
- Unstable coronary artery syndrome
- Age < 18 years
- Inability to comply with the protocol
- Expected survival < 12 months due to any situation other than COPD disease
- Duration of ICU stay > 10 days for this exacerbation
- Not affiliated to national health insurance
- Measure of legal protection (guardianship, wardship, or judicial protection) for patients over the age of majority.

Screen failures (screened patients who finally do not match all criteria) are collected in the eCRF with the reason for non-inclusion.

Recruitment of patients was halted throughout the confinement period of the COVID pandemic 2020. In case of further confinements, the study period will be extended.

### Medical devices used during the study

The medical devices used to study home ventilation strategies will be home ventilators, which are routinely used at home to ventilate patients with chronic hypoventilation (CE-, ISO marked). Consequently, the investigators will be free to use the ventilator of their choosing based on the suitability and comfort for the patient, but ventilators with tools for monitoring the quality of NIV are mandatory.

As during standard care, the patients will receive an educational session at each visit, to be shown how to use the ventilator in case of an acute problem. Devices will be maintained by home providers, who will be available 24 h 7 days a week in case of technical problems in all the 4 countries.

### Informed consent

The participant’s free and informed consent will be obtained in writing by the principal investigator or by a doctor representing the investigator before enrolment into the study. The person will be given sufficient information and time to give an informed consent. To this end, the participant will be handed out the patient information sheet in his native language and a copy of the informed consent form (SI 1), which must be signed in order to be enrolled. If it is physically impossible for the patient to consent in writing, his or her consent will be confirmed by a third party. This third party will have no connection with the investigator or with the sponsor.

The investigator will specify the methods and circumstances of the informed consent in the study participant’s medical file and retain one original of the signed and dated consent form while a second original will be handed to the participant.

### Randomization

Consenting patients matching the inclusion criteria will be randomized to one of the four treatment arms using a centralized, secure, computer-generated, interactive, web-response system accessible from each study center. The randomization will be balanced by blocks of variable and undisclosed size and stratified on the center.

As the effectiveness of blinding has limited scientific justification for this approach, the unblinded trial design and lack of a sham device, with blinded assessment in terms of the outcome, is consistent with other clinical trials in this field [15].

### Study procedure

The study will be performed in accordance with Good Clinical Practice (GCP) guidelines and international and national regulations in force. The study procedures are presented in Table 1.

**Table 1:** Applicability of procedures per study visit. *V0–V8* study visits, *M1–M36* visit time in months, *1D/15D* visit tolerance in days, *1* if not done previously, *2* if follow-up necessary, *3* hand-out of diary; * only for 3 home NIV arms

Visits	VO Baseline	V1 M1±5D	V2 M3 ±15D	V3 M6±15D	V4 M12±15D	V5 M18±15D	V6 M24±15D	V7 M30±15D	V8 M36±15D
Patient information and consent	x								
Inclusion/exclusion criteria	x								
Demographic data	x								
Medical history	x								
Clinical examination									
• Spirometry	x	1/2	1/2						
• 6-minute walk test	x	x	1						
• Diaphragmatic dys function	x								
QoL questionnaires	x	x	x	x	x	x	x	x	x
QoS questionnaires	x	x	x	x	x	x	x	x	x
Dyspnea questionnaire	x	x	x	x	x	x	x	x	x
Charlson comorbidity	x								
Tobacco use	x	x	x	x	x	x	x	x	x
Exacerbations	3	x	x	x	x	x	x	x	x
Laboratory tests									
• Blood gases (PaO2, PaCO2, SpO2)	x	x	x	x	x	x	x	x	x
• PTCO2	x	x	x	x	x	x	x	x	x
• Poly(somno)graphy	x		2						
• Cardiac echography	x		2						
Randomization	x								
Fitting/Settings of ventilator*	x	x	x	x	x	x	x	x	x
Concomitant medication	x	x	x	x	x	x	x	x	x
Device data *		x	x	x	x	x	x	x	x
Adverse events/ hospitalizations		x	x	x	x	x	x	x	x
Educational session	x	x	x	x	x	x	x	x	x

#### Baseline visit

This visit will be conducted by a pulmonologist between 2 days and 2 weeks post normalization of pH following an acute exacerbation and after the results of spirometry and/or polygraphy, if needed. It will include the following procedures:

- Patient information and informed consent
- Verification of inclusion and non-inclusion criteria
- Recording of demographic data
- Recording of personal medical history
  - o No continuous positive airway pressure (CPAP) or home ventilator used at home in the last past 6 months
  - o List of medications (to eliminate more than 3 sedative medications or more than 20 mg of morphine)
  - o No other disease other than COPD with expected survival < 12 months
  - o Assessment of tobacco use (> 10 packs per year)
- Clinical assessment:
  - o Vital status (height, weight, BMI)
  - o Functional respiratory test (spirometry, lung volume, lung diffusion, Pimax, SNIP)
  - o 6 min walk test
  - o Clinical signs of diaphragmatic dysfunction
- Questionnaires
  - o Quality of life (QoL) and quality of sleep (QoS) scores
  - o Dyspnea questionnaire
  - o Charlson comorbidity questionnaire
- Recording of number of exacerbations during the past month
- Laboratory tests
  - o Arterial blood gases (ABG): performed on air > 20′, except if immediate desaturation < 80% or bad tolerance: pH, HCO_3_^−^, PaO2, PaCO_2_, SpO_2_
  - o Nocturnal oximetry with PTCO_2_
  - o Poly (somno)graphy
  - o Cardiac echography (site measurement and central assessment)
- Hand-out of patient diary to follow exacerbations at home and write the treatment received
- Randomization to NIV treatment arm
- Fitting patients with ventilators approved for COPD, ventilator settings, and tolerability
- Setup of NIV in the pulmonology ward for the 3 groups with NIV following last recommendations of setting (high pressure with the aim to decrease PaCO_2_) [14–16]
- Educational session on usage of home ventilation device

#### Follow-up visits M1

This visit will be conducted at the hospital by a pulmonologist at 4 weeks ±5 days after baseline visit. It will include the following procedures:

- Clinical assessment as during baseline visit (spirometry only if not done previously or follow-up necessary)
- Laboratory tests
  - o Arterial blood gases (ABG): performed on air > 20′, except if immediate desaturation < 80% or bad tolerance: pH, HCO_3_^−^, PaO_2_, PaCO_2_, SpO_2_
  - o Nocturnal oximetry with PTCO_2_
- Questionnaires
  - o QoL and QoS scores
  - o Dyspnea questionnaire
- Assessment of tobacco use
- Recording of number of exacerbations since last visit and diary exacerbations assessment
- Concomitant medications
- Recording of adverse events (type, severity, need for hospitalization)
- Recording of hospitalizations (number high-dependency unit (HDU) or ICU, reasons, length of stay)
- Verification if primary endpoint has been reached

For the 3 home NIV treatment arms:

- Device settings
- Hours of utilization (from the device counter)
- Grenoble score for NIV tolerability
- Adjustment of ventilator settings, if necessary and test on the patient
- Educational session on usage of home ventilation device

For the targeted home NIV treatment arm:

- Adjustment of ventilator settings according to algorithm published by Janssens et al. [18]
- Fragmental use of the ventilation (> 3 stops/night) (ventilator software)
- Clinical relevant leaks (median and mean)
- Apnea/hypopnea (AH); if yes, index of the 4 last weeks

#### Follow-up visits M3 to M36 and emergency visits

These visits will be conducted at the hospital by a pulmonologist at the given time point ±2 weeks after baseline visit. It will include the following procedures:

- Clinical assessment as during baseline visit (walk test and spirometry at M3 only if not done previously or follow-up necessary)
- Laboratory tests:
  - o Arterial blood gases (ABG): performed on air > 20′, except if immediate desaturation< 80% or bad tolerance: pH, HCO_3_^−^, PaO_2_, PaCO_2_, SpO_2_
  - o Nocturnal oximetry with PTCO_2_
  - o Poly (somno) graphy (only at M3 if follow-up necessary)
  - o Cardiac echography (site measurement and central assessment) (only at M3 if follow-up necessary)
- Questionnaires
  - o QoL and QoS scores
  - o Dyspnea questionnaire
- Assessment of tobacco use
- Recording of number of exacerbations since last visit and diary exacerbation assessment
- Concomitant medications
- Recording of adverse events (type, severity, need for hospitalization)
- Recording of hospitalizations (number HDU or ICU, reasons, length of stay)
- Verification if primary endpoint has been reached

For the 3 home NIV treatment arms:

- Device settings
- Hours of utilization (from the device counter)
- Grenoble score for NIV tolerability
- Adjustment of ventilator settings according to algorithm, if necessary and test it on the patient
- Educational session on usage of home ventilation device

For the targeted home NIV treatment arm:

- Fragmental use of the ventilation (> 3 stops/night) (ventilator software)
- Clinically relevant leaks (median and mean)
- Apnea/hypopnea (AH); if yes, index of the 4 last weeks

#### End-of-study visits at M36 or time of readmission/death (primary endpoint)

If at M36 primary endpoint has not been reached:

- According to follow-up visits

If primary endpoint reached:

- Recording of primary endpoint
  - o Readmission yes/no; time of readmission
  - o Death yes/no; time of death

### Assessment of treatment

An electronic CRF (eCRF) and a patient diary will be used to collect all data.

#### Primary endpoint: admission-free survival

Admission-free survival is defined as time from randomization to hospital admission in relation with severe exacerbation of COPD or death by any cause, whichever event occurred first. If neither event occurs, time from randomization to the last known follow-up visit will be considered. If withdrawal occurs prior to readmission or death, time from randomization to withdrawal will be considered.

All readmissions of recruited patients to the hospital, including ICU, emergency ward and pulmonary or internal medicine ward admissions will be followed up by the investigators. A COPD-related hospital admission is defined by worsening respiratory symptoms (cough, wheeze, increased sputum production, increased volume of sputum, and/or increased breathlessness), as assessed by the senior physician, leading to treatment for an acute exacerbation of COPD on the day of hospitalization.

Acute NIV will be introduced at pH < 7.35 and PaCO_2_ > 45 mmHg or if RR > 23 persisting after bronchodilators and controlled oxygen therapy (BTS guidelines and Cochrane review 2017). This introduction of NIV is mandatory to fulfill the “readmission” criterion and will be considered as the primary endpoint reached.

#### Secondary endpoints

- Global survival
  - o Overall survival estimated using the Kaplan-Meier method
- Exacerbation frequency at 12 months
  - o The number of patients that experience one or more exacerbations resulting in hospitalization
  - o The number of patients that experience an exacerbation resulting in physician-directed treatment, self-management, or no treatment change
- Comorbidity from baseline to months 1, 3, 6, 12, 18, 24, 30, and 36
  - o Change in arterial partial pressure of carbon dioxide (PaCO_2_)
  - o Change in arterial partial pressure of oxygen (PaO_2_) severe respiratory insufficiency
  - o Disability due to COPD: changes in COPD Assessment Test (CAT)
- Quality of life changes from baseline to months 1, 3, 6, 12, 18, 24, 30, and 36
  - o QoL of respiratory diseases: changes in St George’s respiratory questionnaire, Severe Respiratory Insufficiency Questionnaire (SRIQ) score
  - o QoL with a general score: changes in SF36 and EQ 5D5L
- Sleep changes from baseline to months 1, 3, 6, 12, 18, 24, 30, and 36
  - o QoS: changes in Pittsburgh score and Epworth Sleepiness Scale (ESS)
- Dyspnea
  - o Modified Medical Research Council (MMRC) Dyspnea scores
- Adverse events (AEs) frequency at 3, 6, 12, 18, 24, 30, and 36 months.
  - o Grenoble Stroke and Aphasia Quality of Life (SAQoL) scores
- Serious adverse events (SAEs)
  - o Tympanic membrane perforation, intestinal volvulus, pneumothorax, pneumomediastinum, subcutaneous emphysema, acute glaucoma, cerebrospinal fistula, syncope, hypotension, soreness on the nasal bridge needing stopping NIV more than 7 days, severe epistaxis needing hospitalization
- Cost-effectiveness
  - o Total healthcare costs

### Discontinuation of intervention

The study intervention will be discontinued if any of the following exclusion criteria is present:

- Tracheostomy for other reason than acute respiratory insufficiency
- Facial surgery with contraindication to NIV
- Pulmonary cancer needing surgery
- Pulmonary transplantation
- Pulmonary volume reduction (by surgery, valves or coils)

Furthermore, participants may withdraw from the study without any reason and the investigator might temporarily or permanently withdraw a participant from the study for any safety reason or in the participant’s best interests.

### Adverse event reporting

All adverse events (serious and non-serious) will be recorded in the AE pages of the eCRF. The investigator will assess the seriousness of each adverse event and will thoroughly document serious adverse events, including a definitive medical diagnosis, if possible. The investigator will furthermore assess the intensity (mild, moderate, severe) as well as causal relationship (according to WHO-UMC) between AEs and the study interventions. AEs will be followed up until resolution or stabilization at an acceptable level.

Serious adverse events will additionally be notified to the sponsor without delay, except for those defined by the protocol and investigators brochure as not requiring notification. The notification will be followed by a written report describing the course of the event and providing additional information, as well as any additional anonymized documents. The sponsor will then report all SAEs that are both unexpected and reasonably related to the study intervention to Health Authorities and Ethics Committees of involved countries in accordance with international and local regulations. The sponsor will report all safety information from the trial in the Annual Safety Reports.

### Data management and protection

Data recorded in the eCRF will be identified by a patient number and initials (first letter of last name and first letter of first name). No directly nominative data will be collected and all data sent to the sponsor will be anonymized before transfer.

In accordance with the GCP, the sponsor will ensure data quality by data monitoring and auditing. The persons responsible for these tasks will take all necessary precautions to ensure confidentiality and are bound by professional secrecy. All participants will have agreed to the access of their personal data for quality control in writing.

### Data processing

The eCRF has been developed by CLINFILE. Data will be entered via a secured web interface and stored on a secured platform. Data will be processed in accordance with the provisions of Regulation (EU) 2016/679 (General Data Protection Regulation).

Data will not be shared with third parties, but only between investigators and La Fondation du Souffle.

### Calculation of sample size

With an expected 1-year admission-free survival of 35% in the control group and 55% in the home NIV groups, an accrual period of 2 years, and a minimum follow-up of 12 months, 93 patients in each group are required to provide the study a power of 80% to detect a difference between the control group and each of the three NIV groups, with a familywise error rate (FWER) of 5%. Sample size was estimated by simulations of 200 trials with SAS Version 9.4.

### Statistical analysis

All analyses will be performed on the intent-to-treat (all randomized patients) and total (including non-randomized screen-failures) populations.

All analyses will be performed on the intent-to-treat and total populations. With the exception of the statistical analysis of the primary endpoint, all statistical tests will be performed at the 5% significant level (two-sided formulation).

If applicable, missing data will be taken into account using multiple imputations.

#### Primary endpoint: admission-free survival

Admission-free survival is defined as the time from randomization to hospital admission or death. Each comparison will be done with a two-sided log-rank test.

#### Secondary endpoints: superiority of home NIV

The superiority of any of the three home NIV strategies over the hospital-based NIV in terms of:

- Global survival
- Exacerbation frequency
- Arterial partial pressure of carbon dioxide (PaCO2) and arterial partial pressure of oxygen (PaO2)
- QoL (Charlson, Severe Respiratory Insufficiency Questionnaire score, St George’s Respiratory Questionnaire score, EuroQol, Medical Research Council dyspnea score, Epworth sleepiness score, Pittsburgh score, CAT score, SF36, EQ 5D5L questionnaire)
- Dyspnea
- Costs management
- Serious adverse events (SAEs) occurrence frequency

Analyses will be based on a three-step Bonferroni-based chain procedure. With this procedure, the familywise error rate (FWER) will be controlled.

In the first step, nocturnal NIV and nocturnal NIV with high monitoring will be compared with the control group. Tests will be performed at the two-sided 0.025 level. If no test is significant, no further comparison will be made. If at least one comparison is significant, the rescue NIV group will be compared with the control group at the 0.025 level if only one test was significant at step 1 or at the 0.05 level if both tests were significant. If the comparison between the control group and the rescue NIV group is performed at the 0.025 level and is significant, the not significant comparison at step 1 will be re-tested at the 0.05 level [18].

#### Secondary endpoint: cost-effectiveness

Cost-effectiveness analysis will be analyzed as cost per avoided admission, including:

- Equipment costs
- Maintenance and support costs for the home NIV
- Medical, nursing, and support staff
- Hospital admissions

Economic evaluation of home-based NIV in patients with severe COPD based on (1) the 4-arm comparison and (2) the international population, following the recommendations from the French national health authority and the CHEERS statement [19, 20].

### Insurance

The sponsor has taken out insurance for the full study period, covering its own civil liability and that of any agent (doctor or research staff). The sponsor will also provide full compensation for any damages caused by the study to the study participants and their beneficiaries, unless the sponsor can prove that the harm is not the fault of the sponsor or any agent. Compensation cannot be refused on the grounds of a third party act or the voluntary withdrawal of the person who initially consented to participate in the study.

### Scientific committee

Eleven members from France (workgroup of home NIV of the SPLF: Prof. Gonzalez-Bermejo, Dr. Rabec), Spain (workgroup of home NIV of the SEPAR: Profs. Egea, Masa, Anton, Drs. Diaz-Lobato, Gonzalez, Helli, Lujan, Sancho) and Portugal (workgroup of home NIV of the Portuguese Society of Pulmonology, Prof. Winck) exchanged via monthly emails and 3-monthly workshops email every month in order to determine the objectives and write the protocol. They continue to advise on changes to the protocol throughout the study.

### Steering committee

Composed of 5 members (the 3 national coordinating investigators: Profs. Gonzalez-Bermejo, Anton, Winck; the biostatistician Dr. Hajage; and the sponsor’s representative, Prof. Housset), this committee defined the overall structure of the study and determined the initial methodology. It coordinates information and oversees the study including follow-up on termination and removal rules by monthly email exchange and conference calls upon demand.

### Data Safety Monitoring Board (DSMB)

This study has no specific DSMB as the pharmacovigilance for this type of study is managed locally. The oversight of the data, conduct and progress of the study are performed by the steering committee.

### Audits and inspections

An audit can be carried out at any time by independent persons appointed by the sponsor or the competent authority. The aim of the audits is to ensure the quality of the study, the validity of the results, and compliance with the legislation and regulations in force. The persons leading and monitoring the study agree to comply with the sponsor’s requirements and with the competent authority regarding study audits or inspections. The audit may encompass all stages of the study, from the development of the protocol to the publication of the results and the storage of the data used or produced as part of the study.

## Discussion

Despite COPD being the principal indication of home NIV [21], only three [10, 15, 16] out of six [6–8, 10, 15, 16] randomized controlled studies have shown the benefit of home ventilation. This contradiction leads to completely different rates of implementation of this treatment in different countries with, for example, 1% of COPD patients receiving home NIV in New Zealand [22] and 49% of COPD patients receiving this treatment in Hong Kong [12].

R2M is a novel study on home NIV in COPD patients with the aim to resolve this paradox.

The main objective of this study is to answer the question, if the quality of nocturnal NIV might be responsible for the negative results. This will be achieved by providing a group of patients with extensive monitoring and personalized (targeted) ventilation settings. This entails the major limitation of this study: the risk of providing a treatment based on exceptional expert care which might not be routinely feasible and in all cases.

Furthermore, R2M will investigate the comorbidities typical for COPD patients, such as cardiac insufficiency, OSA, or associated asthma, which might influence ventilation outcomes. To this end, cardiac ultrasound, spirometry, and polysomnography will be performed upon inclusion and before randomization. These extensive evaluations during the 15 days between inclusion and randomization of patients are relatively burdensome, but these examinations are usually routinely recommended.

Additionally, R2M will record the reasons for non-inclusion of patients by a dedicated page in the eCRF, in order to evaluate the percentage of ventilated COPD patients that are screening failures. Indeed, the three studies that have demonstrated the benefit of home ventilation for survival had significant recruiting difficulties with up to 94% of screening failure [10, 15, 16].

The GOLD 2017 document already indicates home NIV to be of some use for a selected group of patients [17], and long-term home non-invasive ventilation (NIV), specifically adapted to the patients “phenotype”, is supposed to improve the prognosis for COPD patients who remain persistently hypercapnic. Therefore, considering the impact of COPD on the patient’s quality of life and life expectancy [12, 13] as well as the GBD [1, 2], the R2M study remains of significant importance despite its relative constraints.

### Trial status

This study is running in France since 09/05/2019 with the first patient having been recruited on 04/07/2019 and an inclusion period of 24 months. The study has equally been submitted in Spain and Portugal on 10/09/2019 and 22/07/2019, respectively. The current version of the protocol is version 4.0, dated May 28, 2019.

## Data Availability

The owner of the data is the sponsor, La Fondation du Souffle. The data cannot be used or disclosed to a third party without its prior permission and upon analysis of the demand of the scientific committee of la Foundation du Souffle. Results of the study will be disseminated in the final report, scientific publications, and in the context of scientific congresses.

## Abbreviations

ABG: Arterial blood gases
AHI: Apnea/hypopnea index
AHRF: Acute hypercapnic respiratory failure (AHRF),
BMI: Body mass index
BTS: British Thoracic Society
CAT: COPD Assessment Test
CE: Conformité Européenne (European Conformity)
CPAP: Continuous positive airway pressure
COPD: Chronic obstructive pulmonary disease
ESS: Epworth Sleepiness Scale
FEV1: Forced expiratory volume in 1 s
FVC: Forced vital capacity
FWER: Familywise error rate
GBD: Global burden of disease
GCP: Good Clinical Practice
GOLD: Global Initiative for Chronic Obstructive Lung Disease
HDU: High-dependency unit
ICU: Intensive care unit
ISO: International Organization for Standardization
MMRC: Modified Medical Research Council
NIV: Non-invasive ventilation
OSA: Obstructive sleep apnea
OSAHS: Obstructive sleep apnea hypopnea syndrome
QoL: Quality of life
QoS: Quality of sleep
SAE: Serious adverse event
SAQoL: Stroke and Aphasia Quality of Life
SRIQ: Severe Respiratory Insufficiency Questionnaire
