# Correction to: Respiratory support in COPD patients after acute exacerbation with monitoring the quality of support (Rescue2-monitor): an open-label, prospective randomized, controlled, superiority clinical trial comparing hospital- versus home-based acute non-invasive ventilation for patients with hypercapnic chronic obstructive pulmonary disease

**DOI:** 10.1186/s13063-020-04900-3

**Published:** 2020-11-23

**Authors:** On behalf of the Rescue2-monitor group

**Affiliations:** 1 Gonzalez-Bermejo, J. Sorbonne Université, INSERM, UMRS1158, Neurophysiologie Respiratoire Expérimentale et Clinique, 75005 Paris, France; 2 AP-HP, Groupe Hospitalier Universitaire APHP-Sorbonne Université, site Pitié-Salpêtrière, Service de Pneumologie, Médecine Intensive et Réanimation, (Département R3S), 75013 Paris, France

**Correction to: Trials 21, 877 (2020)**

**https://doi.org/10.1186/s13063-020-04672-w**

After publication of the original article [1], it came to the authors’ attention that one sponsor declaration was missing in the funding section.

The funding section of the original article has been updated as follows in order to include L3medical as additional sponsor.

Funding: Monetary support for sponsoring the study will be provided by La Fondation du Souffle (ex. BREAS), Sociedad espanola de neumologia y de cirurgia toracica (SEPAR), the Société de pneumologie de langue française (SPLF), L3medical, and Air Liquide Healthcare. La Fondation du Souffle, as sponsor, is involved in the data collection and has ownership of the data. However, it has no involvement in design of the study, analysis, and interpretation of the data and in writing the protocol/manuscript. The SEPAR and SPLF were involved in the design of the study and will be involved in data analysis and interpretation as well as the writing of protocol/manuscripts. Air Liquide Healthcare is not involved in any of the following aspects: study design; collection, management, analysis, and interpretation of data; writing of the protocol or manuscript.

This change does not affect the scientific content of the paper.

The original article has been corrected.
